# Cross-cultural adaptation and psychometric properties of the Iowa-Netherlands Comparison Orientation Measure for the Brazilian context

**DOI:** 10.47626/2237-6089-2022-0573

**Published:** 2024-04-23

**Authors:** Gessyka Wanglon Veleda, Giulia Rodrigues Seoane, Gabriely Ribeiro Ezequiel, Caroline Machado Ferreira, Vera Lúcia Marques de Figueiredo, Tharso de Souza Meyer, Jaciana Marlova Gonçalves Araújo, Luciana Rizo, Taiane de Azevedo Cardoso, Kyara Rodrigues de Aguiar, Luciano Dias de Mattos Souza

**Affiliations:** 1 Programa de Pós-Graduação em Saúde e Comportamento Universidade Católica de Pelotas RS Pelotas Brazil Programa de Pós-Graduação em Saúde e Comportamento, Universidade Católica de Pelotas (UCPel), RS, Pelotas, Brazil.; 2 Faculdade Anhanguera de Rio Grande Rio Grande RS Brazil Faculdade Anhanguera de Rio Grande, Rio Grande, RS, Brazil.; 3 UCPel RS Pelotas Brazil UCPel, RS, Pelotas, Brazil.; 4 Universidade Federal do Rio Grande Rio Grande RS Brazil Universidade Federal do Rio Grande, Rio Grande, RS, Brazil.; 5 Programa Inter-Universitário de Doutoramento em Psicologia na Especialidade de Psicologia da Educação Universidade de Coimbra Coimbra Portugal Programa Inter-Universitário de Doutoramento em Psicologia na Especialidade de Psicologia da Educação, Universidade de Coimbra, Coimbra, Portugal.; 6 Department of Psychiatry and Behavioural Neurosciences McMaster University Hamilton ON Canada Postdoctoral Fellow, Department of Psychiatry and Behavioural Neurosciences, McMaster University, Hamilton, ON, Canada.; 7 Programa de Pós-Graduação em Psiquiatria e Ciências do Comportamento Departamento de Psiquiatria Universidade Federal do Rio Grande do Sul Porto Alegre RS Brazil Programa de Pós-Graduação em Psiquiatria e Ciências do Comportamento, Departamento de Psiquiatria, Universidade Federal do Rio Grande do Sul, Porto Alegre, RS, Brazil.

**Keywords:** INCOM, cross-cultural adaptation, validity, reliability, factor analysis

## Abstract

**Introduction:**

The Iowa-Netherlands Comparison Orientation Measure (INCOM) was developed to measure individual differences in social comparison orientation and has been widely used in research and various different settings.

**Objectives:**

The aim of this study was to adapt the online version of the INCOM and to evaluate its psychometric parameters when applied to a Brazilian population of university students.

**Methods:**

The procedures were divided into two steps: step 1 – cross-cultural adaptation and analysis of content validity, and step 2 – assessment of psychometric characteristics. Step 1 comprised the processes of translation, evaluation by an expert committee, evaluation by the target population, and back-translation. For step 2, 1,065 university students were recruited and then factor analysis, analysis of reliability, and analysis of validity based on external measures were performed.

**Results:**

The adaptation process yielded satisfactory results, including good indicators of content validity. Exploratory factor analysis revealed a two-dimensional structure and adequate factor loadings, except for item 11, which was excluded from the final version. Additionally, the final version of the scale had adequate fit indices (χ^2^ = 148.45, degrees of freedom [df] = 26; p < 0.001; root mean square error of approximation [RMSEA] = 0.06; comparative fit index [CFI] = 0.99; and Tucker-Lewis index [TLI] = 0.98). Evidence of reliability (Cronbach’s alpha = 0.83) was observed and there were positive correlations with negative affect (r = 0.36) and negative correlations with positive affect and self-esteem (r = -0.15; r = -0.41, respectively).

**Conclusion:**

The Brazilian version of the INCOM presents satisfactory psychometric parameters and can thus be used to measure social comparison orientation.

## Introduction

According to Festinger’s theory of social comparison processes,^1^ all subjects have an impulse to evaluate their abilities and opinions in comparison to others, especially as objective and non-social means are not available. Although all subjects engage in social comparisons, the extent to which they do so may vary from one individual to another.^2-4^

To measure these individual differences, Gibbons and Buunk^5^ constructed the Iowa-Netherlands Comparison Orientation Measure (INCOM), which assesses social comparison orientation, according to Festinger’s theory.^1^ The INCOM was simultaneously developed for the American and Dutch populations and comprises 11 items distributed between two factors: abilities and opinions.

The original scale presents satisfactory psychometric parameters, with good fit indices (χ^2^ = 520.2, degrees of freedom [df] = 1; p < 0.001) and adequacy index (GFI), and adjusted GFI (AGFI) are > 0.95 for both factors.^5^ There is also growing evidence of convergent validity, based on moderate and strong correlations with competing measures, such as the Attention to Social Comparison Information (ATSCI)^6^ scale (Dutch samples: r = 0.66 and American samples: r = 0.47), in addition to satisfactorily predicting the behavior of social comparison in four experimental studies.^5^

Gibbons and Buunk^5^ also found significant correlations between social comparison and negative affectivity, with higher negative affect scores (Positive and Negative Affect Schedule [PANAS^7^]: r = 0.39, Dutch samples, and r= 0.29, American samples), lower self-esteem (Rosenberg Self-Esteem Scale^8^ [RSE]: r = -0.32, Dutch samples, and r = -0.18, American samples), and greater neuroticism (Netherlands Personality Questionnaire^9^: r = 0.37, Dutch samples, and r = 0.33, American samples). The scale shows evidence of reliability, with a 0.8 Cronbach’s alpha in the original sample and temporal stability of 0.60 for re-administration after 1 year in the United States and of 0.72 after 7.5 months in the Dutch sample.^5^ It is noteworthy that the INCOM did not present significant correlations with measures of social desirability,^5,10^ an especially important characteristic since social comparisons can be considered inadequate and associated with non-valued characteristics, such as helplessness and lack of autonomy.^5,11^

The INCOM has been widely adapted for other countries, such as Germany,^11^ Russia,^12^ Portugal,^13,14^ Spain,^15,16^ and Chile,^16^ with equally satisfactory psychometric parameters. Furthermore, its field of application seems to be extensive and complex, since social comparison has been associated with different dimensions of work,^17^ well-being,^18^ depression and anxiety,^19^ use of social networking sites,^20^ and body satisfaction,^21^ for example.

Given the wide use of the INCOM and its adequate psychometric parameters, there is an evident need to adapt it for Brazil, enabling measurement of social comparison in this population. Thus, the aim of this study was to adapt the online version of the INCOM and to evaluate its psychometric parameters when administered to a Brazilian population of university students.

## Methods

**Figure 1:**
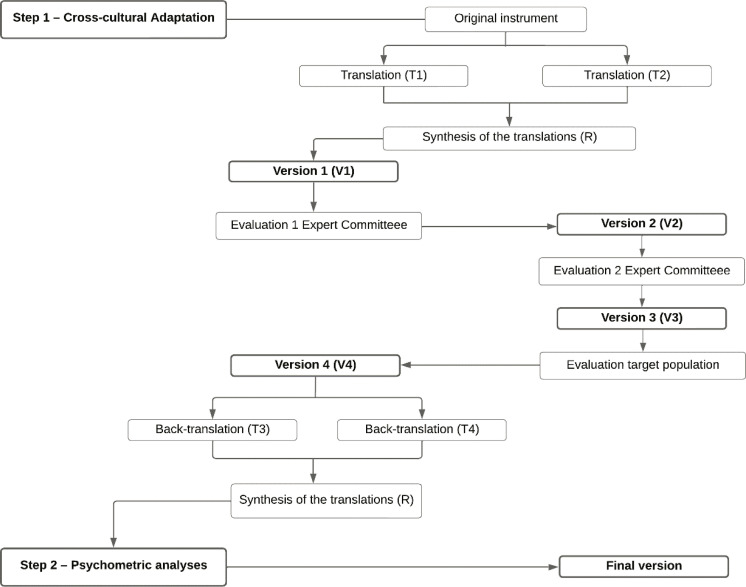
Methodological procedures of the cross-cultural adaptation and psychometric analyses of the Iowa-Netherlands Comparison Orientation Measure (INCOM) scale (steps 1 and 2).

This study was carried out based on the instrument cross-cultural adaptation method proposed by the International Test Commission (ITC),^22^ Borsa et al.,^23^ and Pasquali,^24^ and was approved by the research ethics committee (CAAE – step 1: 47946621.3.0000.5339; CAAE – step 2: 47931821.2.0000.5339). The procedures performed were divided into two steps: step 1 – cross-cultural adaptation and content validity, and step 2 – assessment of psychometric characteristics in a cross-sectional study. Figure 1 presents the two different steps and their methodological approaches.

All data collection was carried out online, following the guidelines of Circular Letter number 2 of February 24, 2021, from the National Research Ethics Committee, covering research procedures in a virtual environment.^25^ All participants (steps 1 and 2) agreed to an informed consent statement.

### Step 1 – Cross-cultural adaptation and content validity

This step aimed to perform the translation and cross-cultural adaptation of the scale, as well as to measure its content validity and validity based on the response process. Prior to the translation process, the authors of the scale were contacted and authorized its adaptation for the Brazilian context. The original scale was therefore sent to two independent translators (T1 and T2), one with knowledge about the construct and the other a sworn translator. Both were fluent in Portuguese and had a high level of fluency in English. The researchers (R) (GWV and LDMS) conducted a synthesis of the two translated versions, evaluating their semantic, idiomatic, conceptual, linguistic, and contextual discrepancies,^23^ arriving at a single version (V1).

V1 was forwarded to an expert committee comprising four psychologists with technical knowledge in psychometry and/or in the construct under evaluation. After the first evaluation by the committee, new adjustments were made to the scale resulting in version 2 (V2). This version was resubmitted for expert evaluation and again underwent minor adjustments (V3). To measure the content validity, the expert committee evaluated the scale, scoring it from 1 to 5 for five Likert-type questions rating language clarity and its theoretical dimension. These results were used to calculate the content validity coefficient (CVC).^26^ The evaluation also included a descriptive assessment of the scale, with a space for suggestions and modifications, which were qualitatively evaluated by the researchers (GWV and LDMS).

With V3, the step of evaluation by the target population began, in which 11 university students participated. Seven (64%) of the participants were women and the average age of the sample was 22.1 years. Participants answered the INCOM online and evaluated it in response to four questions rating clarity, adequacy, and understanding of the scale; measured on a Likert scale ranging from 1 to 5 points. These results were used to calculate a CVC for the target population.^26^ To measure the validity based on the response process of the scale,^27^ two synchronous remote focus groups were assembled, with four and five university students respectively, to observe how participants responded to the scale and its involved processes, in addition to allowing greater detailing of the suggested indications.

After adjustments, a fourth version of the scale (V4) was completed and was sent for back-translation. This process was performed by two translators (T3 and T4), different from the first translators, one a bilingual psychologist and the other a professional specialist in translation processes. Once more, the researchers (GWV and LDMS) performed a synthesis of the back-translations and forwarded this version to the original authors of the scale, who approved it without suggesting further changes.

### Step 2 – Assessment of psychometric characteristics

This step aimed to gather evidence of validity based on the internal structure, reliability, and validity based on the relationship with external measures. For the sample calculation, a recommendation available in the literature for the process of cross-cultural adaptation of mental health instruments was considered.^24^ The recommendation is an approximate sample size of 10 subjects per item or 100 per factor/instrument dimension. Assuming the scale’s two dimensions, the recommended sample size would be 200 subjects. However, this step is part of a larger study called “Does the use of Instagram, mediated by social comparison and self-esteem, impact the affect of Brazilian university students?,” which aims to longitudinally verify the relationships between social comparison, self-esteem, positive and negative affects, and the intensity and profile of use of the social network Instagram by university students in Brazil. A sample calculation indicated a sample of 940 subjects and a total of 1,065 participants were recruited. It is worth mentioning that the increased sample size does not affect the study objective, since it is recommended that for verification of psychometric parameters the samples should be large enough to enable the availability of statistical information.^22^

A total of 1,065 Brazilian university students from all regions of the country participated in this stage. Most of them were women (68.8%), white-skinned (56.1%), aged between 18 and 64 years (mean = 23.4, standard deviation [SD] = 6.1), enrolled in courses from the human sciences (29.7%), and did not report the presence of a psychiatric diagnosis at the time (79.2%). Only undergraduate students were included. Sample selection employed a non-probabilistic method, but we sought to recruit participants in a stratified manner, according to the region of Brazil in which they resided (South, Southeast, Midwest, Northeast, and North). Thus, the sample was drawn 41% from the Southeast, 20.2% from the South, 20% from the Northeast, 10.2% from the Midwest, and 8.5% from the North, in similar proportions to the relative distribution frequency of university students in the country, based on data provided by the Brazilian Ministry of Education^28^ (MEC).

Sample recruitment was carried out via the internet and the invitation with the link to access the questionnaire was sent using several different platforms, such as e-mail, Instagram, and WhatsApp.

### Instruments

#### General questionnaire

A structured questionnaire containing variables regarding sex, age, state of residence, institution, course, and current presence of psychiatric diagnosis (self-report).

#### Iowa-Netherlands Comparison Orientation Measure^5^ (INCOM)

A scale developed to measure individual differences in comparative orientation, that is, an individual’s inclination to collect information about other people and/or to compare information for their own assessment. The INCOM comprises 11 items divided between two factors. The first factor concerns comparison of abilities and includes six items related to performance, which indicate “how skilled am I compared to others?” The second factor, referring to opinions, encompasses the five remaining items, associated with “what should I think?” or “how should I feel?” based on the comparison with others. Response options vary along a Likert scale from (1) “I disagree strongly” to (5) “I agree strongly,” although questions 5 and 11 are scored in reverse. Higher scores indicate that the subject is more likely to collect information about other people and/or to apply that information to their own situations. In this phase of the project, the version of the scale already semantically adapted to the Brazilian context was applied, maintaining all 11 items (V4).

#### Positive and Negative Affect Schedule7 (PANAS)

A self-report instrument containing two subscales and a total of 20 items designed to measure positive and negative affect. These are conceptualized as distinct dimensions of emotional experience. Positive affect is related to experiencing positive mood, with feelings such as interest, and enthusiasm. Meanwhile, negative affect is associated with emotions such as nervousness, fear, and guilt. It has a Likert-type response scale ranging from (1) “very slightly or not at all” to (5) “extremely,” identifying the extent to which the respondent has experienced each emotion in the last few days. The scale provides two independent scores, one for positive affect and one for negative affect.

The PANAS is one of the most widely used instruments for measuring affect and has been adapted and validated with good psychometric results for several countries,^29-36^ including Brazil,^33-35^ The results of the most recent study indicate that the PANAS has satisfactory psychometric properties, with Cronbach’s alpha = 0.84 for the positive affect scale and 0.90 for the negative affect scale.^35^ In this study, positive and negative affect scores were used to measure negative and positive convergent validity, respectively, with the INCOM scale. In the current sample, the scale maintained satisfactory reliability parameters with Cronbach’s alpha coefficients of 0.92 and 0.91 for the positive and negative affect subscales, respectively.

#### Rosenberg Self-Esteem Scale^8^,^37^ (RSES)

This is a one-dimensional measure that globally assesses self-esteem from 10 statements based on a set of feelings related to self-esteem and self-acceptance. Responses are provided on a Likert scale ranging from (0) “strongly disagree” to (4) “strongly agree” and the higher the score the greater the self-esteem.

This has become one of the most widely used instruments for assessment of self-esteem, having been translated into 28 languages and distributed in more than 53 countries.^38^ In Brazil, this instrument was originally adapted and validated for research by Hutz^39^ and revalidated by Hutz and Zanon,^37^ with satisfactory psychometric properties, presenting Cronbach’s alpha = 0.90. In this study, self-esteem scores were used to measure negative convergent validity with the INCOM scale. The RSES maintained satisfactory reliability parameters for the current sample, with Cronbach’s alpha = 0.90.

#### Statistical analyses

The data obtained in step 1 were analyzed using Microsoft Office Excel software. The cut-off point adopted for the CVC was ≥ 0.80, for each of the items and also for the overall score.^24^

In step 2, the statistical programs FACTOR and the Statistical Package for Social Sciences (SPSS 22.0) were used. An Exploratory Factor Analysis was performed aiming to evaluate the factor structure of the INCOM. The analysis was implemented using a polychoric matrix and the robust diagonally weighted least squares^40^ (RDWLS) extraction method. The number of factors to be retained was determined using the parallel analysis technique with random permutation of the observed data^41^ and the Robust Promin rotation method.^42^

The scale’s unidimensionality was investigated using the indicators unidimensional congruence (UniCo), explained common variance (ECV), and the mean of item residual absolute loadings (MIREAL). Values of UniCo < 0.95, ECV < 0.85, and MIREAL > 0.30 indicate that unidimensionality is not supported.^43^

The fit of the model was evaluated using the root mean square error of approximation (RMSEA), the comparative fit index (CFI), and the Tucker-Lewis index (TLI) fit indices. According to literature,^44^ RMSEA values should be < 0.08, with a confidence interval not reaching 0.10, whereas CFI and TLI values must be > 0.90 or preferably > 0.95.

The stability of the factors was assessed using the H index. The H index assesses how well a set of items represents a common factor. H values range from 0 to 1. H values > 0.80 suggest a well-defined latent variable, which is more likely to be stable across different studies. Low values of H suggest an ill-defined latent variable, and probable instability across different studies.^43^

Moreover, the factor determinacy index (FDI), the overall reliability of fully-informative prior oblique N-EAP scores (ORION), the sensitivity ratio (SR), and the expected percentage of true differences (EPTD) were also considered. These indices assess the quality and accuracy of factor score estimates, indicating scale adequacy for both research applications and individual clinical assessments. For this, the values recommended are as follows: FDI > 0.90, ORION > 0.80, SR > 2, and EPTDs > 90%.^43^

The scale’s reliability indices were also evaluated using composite reliability and Cronbach’s alpha, with values > 0.70^45^ being considered adequate. For the validity based on the relationships with external measures, the associations between the INCOM score and positive and negative affect (PANAS) and self-esteem (RSES) were calculated using the Spearman correlation test,^46^ due to the non-parametric distribution of continuous variables. Correlations with p < 0.05 were considered statistically significant.

## Results

### Step 1 – Cross-cultural adaptation and content validity

Table 1 presents the versions of the INCOM scale over the course of the cross-cultural adaptation process, starting with the original scale, followed by the synthesis of the translations (V1), the adjustments after the first and second evaluation by the expert committee (V2 and V3), the changes discussed with the target population (V4), the back-translation, and the final version.

**Table 1:** Comparison between the original Iowa-Netherlands Comparison Orientation Measure (INCOM) scale and the different versions over the course of the cross-cultural adaptation process

Original version	Version 1	Version 2	Version 3	Version 4	Back-translated version	Final version
1. I often compare how my loved ones (boy or girlfriend, family members, etc.) are doing with how others are doing.	Comparo como as pessoas que amo (namorado(a), familiares, etc.) estão em relação a como outras pessoas estão.	* **Eu frequentemente** comparo como as pessoas que amo (namorado(a), familiares etc.) estão em relação a como outras pessoas estão.	*Eu frequentemente comparo como **estão** as pessoas que amo (namorado(a), familiares etc.) **com como** outras pessoas estão.	*Eu frequentemente comparo como estão as pessoas que amo (namorado(a), familiares etc.) com como **estão outras pessoas**.	I often compare the people I love (partner, family, etc.) to other people.	Eu frequentemente comparo como estão as pessoas que amo (namorado(a), familiares etc.) com como estão outras pessoas.
2. I always pay a lot of attention to how I do things compared with how others do things.	Presto muita atenção em como faço as coisas comparado a como os outros fazem as coisas.	*Eu sempre presto muita atenção em como faço as coisas comparado a como os outros fazem as coisas.	*Eu sempre presto muita atenção em como faço as coisas, comparado **ao modo** como os outros fazem as coisas.	Eu sempre presto muita atenção em como faço as coisas, comparado ao modo como os outros fazem as coisas.	I always pay a lot of attention to how I do things, comparing to how other people do things.	Eu sempre presto muita atenção em como faço as coisas, comparado ao modo como os outros fazem as coisas.
3. If I want to find out how well I have done something, I compare what I have done with how others have done.	Se quero saber o quão bem fiz algo, comparo o que eu fiz em relação a como os outros fizeram.	Se quero saber o quão bem fiz algo, comparo o que eu fiz em relação a como os outros fizeram.	*Se quero saber o quão bem fiz algo, comparo o que eu fiz **a como** os outros fizeram.	Se quero saber o quão bem fiz algo, comparo o que eu fiz a como os outros fizeram.	If I want to know how well I have done something, I compare what I have done to how others have done it.	Se quero saber o quão bem fiz algo, comparo o que eu fiz a como os outros fizeram.
4. I often compare how I am doing socially (e.g., social skills, popularity) with other people.	Comparo como estou me saindo socialmente (por exemplo, habilidades sociais, popularidade) com outras pessoas.	*Eu frequentemente comparo como estou me saindo socialmente (por exemplo, **manifestar opinião, iniciar e manter conversas**, popularidade) com outras pessoas.	Eu frequentemente comparo como estou me saindo socialmente (por exemplo, manifestar opinião, iniciar e manter conversas, popularidade) com como outras pessoas estão.	*Eu frequentemente comparo **minha vida social** (por exemplo, manifestar opinião, iniciar e manter conversas, popularidade) **com a dos outro**.	I often compare my social life (e.g., expressing my opinion, starting and maintaining conversations, popularity) with that of others.	Eu frequentemente comparo minha vida social (por exemplo, manifestar opinião, iniciar e manter conversas, popularidade) com a dos outro.
5. I am not the type of person who compares often with others. (reverse scored)	Não sou o tipo de pessoa que costuma se comparar com outros.	*Não sou o tipo de pessoa que costuma se comparar **frequentemente** com **os** outros.	Não sou o tipo de pessoa que costuma se comparar frequentemente com os outros.	Não sou o tipo de pessoa que costuma se comparar frequentemente com os outros.	I am not the type of person who often compares myself with others.	Não sou o tipo de pessoa que costuma se comparar frequentemente com os outros.
6. I often compare myself with others with respect to what I have accomplished in life.	Me comparo com outros no que diz respeito ao que realizei na vida.	*Frequentemente me comparo com **outras pessoas** no que diz respeito ao que realizei na vida.	Frequentemente me comparo com outras pessoas no que diz respeito ao que realizei na vida.	*Frequentemente me comparo com **os outros** no que diz respeito **as minhas conquistas pessoais**.	I often compare myself with others in what concerns my achievements.	Frequentemente me comparo com os outros no que diz respeito as minhas conquistas pessoais.
7. I often like to talk with others about mutual opinions and experiences.	Gosto de conversar sobre opiniões e experiências mútuas.	*Eu frequentemente gosto de conversar **com outras pessoas** sobre opiniões e experiências **em comum**.	Eu frequentemente gosto de conversar com outras pessoas sobre opiniões e experiências em comum.	Eu frequentemente gosto de conversar com outras pessoas sobre opiniões e experiências em comum.	I often like to talk to other people about common opinions and experiences.	Eu frequentemente gosto de conversar com outras pessoas sobre opiniões e experiências em comum.
8. I often try to find out what others think who face similar problems as I face.	Tento descobrir o que pessoas que enfrentam problemas parecidos com os meus pensam.	*Eu frequentemente tento descobrir **o que pensam as pessoas** que enfrentam problemas parecidos com os meus.	Eu frequentemente tento descobrir o que pensam as pessoas que enfrentam problemas parecidos com os meus.	*Eu frequentemente tento descobrir o que **as pessoas com problemas parecidos com os meus pensam**.	I often try to find out what people with problems similar to mine think.	Eu frequentemente tento descobrir o que as pessoas com problemas parecidos com os meus pensam.
9. I always like to know what others in a similar situation would do.	Gosto de saber o que outras pessoas fariam em uma situação parecida a minha.	*Sempre gosto de saber o que outras pessoas fariam em uma situação parecida **com** a minha.	Sempre gosto de saber o que outras pessoas fariam em uma situação parecida com a minha.	*Sempre gosto de saber o que **outra(s) pessoa(s) faria(m)** em uma situação parecida com a minha.	I always like to know what other people would do in a situation similar to mine.	Sempre gosto de saber o que outra(s) pessoa(s) faria(m) em uma situação parecida com a minha.
10. If I want to learn more about something, I try to find out what others think about it.	Se eu quero aprender mais sobre algo, tento descobrir o que os outros pensam sobre o assunto.	Se eu quero aprender mais sobre algo, tento descobrir o que os outros pensam sobre o assunto.	Se eu quero aprender mais sobre algo, tento descobrir o que os outros pensam sobre o assunto.	Se eu quero aprender mais sobre algo, tento descobrir o que os outros pensam sobre o assunto.	If I want to learn more about something, I try to find out what others think about it.	Se eu quero aprender mais sobre algo, tento descobrir o que os outros pensam sobre o assunto.
11. I never consider my situation in life relative to that of other people. (reverse scored)	Nunca levo em consideração a minha situação de vida em relação à de outras pessoas.	Nunca levo em consideração a minha situação de vida em relação à de outras pessoas.	Nunca levo em consideração a minha situação de vida em relação à de outras pessoas.	Nunca levo em consideração a minha situação de vida em relação à de outras pessoas.	I never consider my life situation in relation to other people.	Item excluded after psychometric analyses.

* Items altered after evaluation, specifically the words in bold.

After synthesis of the translations, the experts’ evaluation indicated a need for language adjustments, especially insertion of adverbs of time and manner (“always,” “often”), in addition to small changes in sentence structure. After these adjustments, a second evaluation was requested by the committee, resulting in satisfactory CVC values of 0.86 for the full scale and > 0.80 for each item.

Version 3, evaluated by the target audience, also presented a satisfactory CVC for all items (> 0.80). During the two focus groups, participants recommended changes to assist in understanding of the items, which included changes in terminology (for example, changing “what I accomplished in life” to “my achievements”) and sentence structure, resulting in version 4 of the scale. It was found that the participants had similar and plausible processes to answer the scale. Thus, these indicators constitute evidence of content validity and validity based on the response processes of the scale.^24,26,27^

### Step 2 – Assessment of psychometric characteristics

With regard to the exploratory factor analysis, Bartlett’s test of sphericity (491.4, df = 55, p < 0.001) and the Kaiser-Meyer-Olkin (KMO) (0.86) test of sampling adequacy both suggested the correlation matrix of the items was interpretable. Table 2 shows the item factor loadings from the exploratory factor analysis. All items had high factor loadings for their respective factors, except for item 11, which had loadings less than 0.30 for both factors, suggesting it should be excluded.^47^

**Table 2:** Structure and factor loadings of versions of the Iowa-Netherlands Comparison Orientation Measure (INCOM) with 11 and 10 items, based on the exploratory factor analysis

Items	INCOM-11		INCOM-10	

	Factor 1 – Abilities	Factor 2 – Opinions	Factor 1 – Abilities	Factor 2 – Opinions
1. Eu frequentemente comparo como estão as pessoas que amo (namorado(a), familiares etc.) com como estão outras pessoas.	**0.52**	0.06	**0.52**	0.05
2. Eu sempre presto muita atenção em como faço as coisas, comparado ao modo como os outros fazem as coisas.	**0.73**	0.09	**0.74**	0.08
3. Se quero saber o quão bem fiz algo, comparo o que eu fiz a como os outros fizeram.	**0.75**	0.13	**0.76**	0.11
4. Eu frequentemente comparo minha vida social (por exemplo, manifestar opinião, iniciar e manter conversas, popularidade) com a dos outros.	**0.82**	-0.02	**0.82**	-0.02
5. Não sou o tipo de pessoa que costuma se comparar frequentemente com os outros.	**-0.78**	0.20	**-0.78**	0.19
6. Frequentemente me comparo com os outros no que diz respeito as minhas conquistas pessoais.	**0.77**	0.03	**0.78**	0.03
7. Eu frequentemente gosto de conversar com outras pessoas sobre opiniões e experiências em comum.	-0.21	**0.65**	-0.21	**0.66**
8. Eu frequentemente tento descobrir o que as pessoas com problemas parecidos com os meus pensam.	-0.05	**0.86**	-0.06	**0.86**
9. Sempre gosto de saber o que outra(s) pessoa(s) faria(m) em uma situação parecida com a minha.	0.02	**0.83**	0.02	**0.84**
10. Se eu quero aprender mais sobre algo, tento descobrir o que os outros pensam sobre o assunto.	0.04	**0.60**	0.04	**0.61**
*11. Nunca levo em consideração a minha situação de vida em relação à de outras pessoas.	-0.23	0.06		

Values in bold indicate the highest factor loading.

* Item with factor loading < 0.30 for the two factors.

Next, the exploratory factor analysis was performed again with the 10-item version of the scale (INCOM-10). In this version, the factor loadings of all items remained high in their respective factors (Table 2), with satisfactory fit indices (χ^2^ = 148.45, df = 26; p < 0.001; RMSEA = 0.06; CFI = 0.99; TLI = 0.98).^42^ Therefore, all subsequent analyses were conducted with the final version of the scale, containing 10 items.

Table 3 shows the result of a parallel analysis, which indicated that two factors of the real data present a higher percentage of explained variance than the random data, suggesting both dimensions of the scale should be retained. When considering the 95% confidence interval (95%CI), factor 2 has a small difference, with the value for real data smaller than the random data. For confirmation purposes, the values of UniCo (< 0.95), ECV (< 0.85), and MIREAL (> 0.30) were calculated and did not support unidimensionality of the scale and so the two-factor structure was therefore maintained.^42^

**Table 3:** Parallel analysis and indicators of unidimensionality for the Iowa-Netherlands Comparison Orientation Measure (INCOM) 10-item version

Factors	Percentage of variance explained of real data	Percentage of variance explained of random data	Percentage of variance explained of random data (95%CI)
1	51.62*	20.33	25.80
2	19.50*	17.83	21.91
3	8.07	15.41	18.20
4	6.70	13.21	15.50
5	5.10	11.05	13.20
6	4.30	8.80	11.30
7	2.50	6.61	9.43
8	1.83	4.50	7.25
9	0.45	2.30	4.91
UniCo (95%CI)	0.87 (0.85-0.90)		
ECV (95%CI)	0.75 (0.73-0.77)		
MIREAL (95%CI)	0.35 (0.33-0.36)		

95%CI = 95% confidence interval; ECV = explained common variance; MIREAL= mean of item residual absolute loadings; UniCo = unique unidimensional congruence.

* Number of factors to be retained.

The H-index replicability measure of the factor structure presented values > 0.80 for both factors (H-latent: factor 1 = 0.90; factor 2 = 0.87; H-observed: factor 1 = 0.88; factor 2 = 0.84), suggesting that this structure may be replicable in future studies. Regarding the quality and precision of the factor score indices, FDI (factor 1 = 0.95; factor 2 = 0.93), ORION (factor 1 = 0.90; factor 2 = 0.87), SR (factor 1 = 2.97; factor 2 = 2.60), and EPTD (factor 1 = 92.4%; factor 2 = 91.2%) all showed adequate results, indicating the scale is also applicable for individual clinical assessment.

For reliability analyses, composite reliability and Cronbach’s alpha were measured for the versions of the scale containing 10 (INCOM-10) and 11 items (INCOM-11). The composite reliability was only adequate for the INCOM-10 version (abilities = 0.88; opinions = 0.80) since the value for the second factor of the INCOM -11 was unsatisfactory (abilities = 0.88; opinions = 0.75). Cronbach’s alpha values were also adequate, slightly higher for INCOM-10 (alpha = 0.83) than for INCOM-11 (alpha = 0.82).

Regarding validity based on external measures, a significant positive correlation was found between the INCOM and the negative affect subscale, and significant negative correlations were found between the INCOM and the positive affect subscale and the RSE (Table 4). Although results were approximate, the correlations between the INCOM-10 and positive affect and self-esteem were significantly higher than the correlations between the INCOM-11 and these scales, as indicated by the Fisher r-to-z transformation test (PANAS – positive affect: z = -5.44; p < 0.001; EAR: z = -3.44; p < 0.00). Thus, the INCOM-10 has a stronger association with external measures than the INCOM-11.

**Table 4:** Correlations between the 10-item and 11-item versions of the INCOM and external variables

Variables	INCOM-10	INCOM-11
PANAS		
Negative affect	0.36	0.34
Positive affect	-0.15	-0.15
RSE	-0.41	-0.39

INCOM =Iowa-Netherlands Comparison Orientation Measure; PANAS = Positive and Negative Affect Schedule; RSE = Rosenberg Self-esteem scale.

All correlations were significant to p < 0.001.

## Discussion

This study achieved its objective of adapting the INCOM scale to the Brazilian context and gathering evidence of its validity. Step 1 included rigorous and systematic processes of translation, back-translation, and evaluation by a committee of experts and by the target audience, in order to guarantee the equivalence of the scale’s content.^22-24^ Satisfactory CVC values together with the qualitative assessments of the processes contribute significant evidence of content validity, enabling verification of other psychometric parameters in the Brazilian context.^24^

In common with the original version, our adaptation maintained the two-factor structure with the same division between the items. The authors of the original scale recognize that a single-factor model also showed acceptable, although less robust, fit indices.^5^ Moreover, using the Mokken analysis, Buunk, et al.^16^ also found a unique, factor structure for the Spanish version of the INCOM (INCOM -E^15^) when administered to a new sample in Spain.

In our study, the parallel analysis indicated a small discrepancy in factor 2 when considering the 95%CI, which may indicate greater fragility of the opinions factor in comparison to the abilities factor. However, none of the unidimensionality indicators were corroborated. Moreover, the fit indices for two factors presented satisfactory results together with the H-index and evidence of the quality and precision of the factors’ scores estimates, indicating the scale is also applicable to the clinical context.^42^

Other adaptations carried out for German,^11^ Portuguese,^13,14^ Spanish,^15^ and American^48^ populations also found a two-factor structure for the INCOM. In view of this evidence, the final version of the INCOM scale adapted to the Brazilian context maintains two factors; the first reflecting an interest in comparison related to performance or ability (items 1 to 6), while the second indicates an interest in comparison based on opinions (items 7 to 10), in consonance with the discussions initiated by Festinger^1^ on social comparison processes.

As for the factor loadings, all items except for item 11 showed satisfactory results, supporting its exclusion from the final INCOM version. Other adaptations have also found that this item malfunctioned. Schneider and Schupp^11^ excluded the same item from the German version of the scale, also due to insufficient factor loading. Chilean and Spanish versions^16^ removed both item 11 and item 5, which is also inverted, due to their factor loadings. Furthermore, exclusion of these items improved the scale’s fit indices. The same happens with specific samples, such as the Portuguese version of the INCOM applied to parents of children with chronic health conditions,^14^ which also excluded both inverted items (items 5 and 11). In adaptations in which item 11 was retained,^13,15,48^ its factor loadings assigned it to the abilities dimension, in contrast to what the original scale proposed (in which it belonged to the opinions dimension), suggesting incompatibilities in its structure.

It is worth mentioning that item 11 was not changed in terms of sentence structure or terminology during step 1. Thus, the unsatisfactory factor loading would not be associated with divergences and specific difficulties in semantic adaptation. Hypotheses of acquiescence to or misunderstanding of inverted items are also not justified, since item 5 had a high factor loading onto its corresponding factor. Thus, item 11 does not seem to be representative for assessment of the proposed content, being excluded from the final version of the scale, which maintained its satisfactory psychometric properties.

Regarding reliability of the scores, both composite reliability^44^ and Cronbach’s alpha presented satisfactory values, very close to the original version of the scale (Cronbach’s alpha = 0.83).^5^ Furthermore, removal of item 11 increased the parameter values, corroborating its exclusion.

The associations with external measures presented the expected directions, similar to those found for the original scale, with moderate correlations^46^ between the INCOM and negative affectivity, with higher negative affect scores and lower self-esteem scores. When adapting the INCOM-E scale, Buunk et al.^15^ also found negative correlations with the RSE. The prototypical image developed by Gibbons and Buunk^5^ indicates that subjects with high social comparison scores present a combination of high accessibility and self-awareness, interest in what others feel and think, and some degree of self-uncertainty and negative affectivity. All of these strengthen the convergent validity of the evaluated instrument.

The association with positive affect was negative and weak, similar to the original scale, which showed weak correlations with the same subscale and with other measures of positive outcome, such as optimism and well-being.^5^ The Spanish adaptation also found weak associations with optimism and psychological well-being.^15^ Thus, the INCOM discriminates negative affect more robustly than positive affect. It is noteworthy that two studies found neuroticism to be the characteristic most associated with social comparison scores.^5,15^ In the original scale, the commonality analysis indicated that the positive relationships between social comparison and other negative affective traits were attributable to its relations with neuroticism.^5^

Our study did not include neuroticism in the measurement of negative convergent validity, but we suggest it should be evaluated in future studies and that the relationship between social comparison and other personality characteristics should also be investigated in order to broaden understanding of the construct. Another limitation concerns the non-systematic sampling process, however, in addition to our sample being large; it also included university students from all regions of the country, contributing to the representativeness of this population. Furthermore, future adaptations in populations with specific characteristics and lower educational levels (clinical samples, for example) could increase the validity of the scale. Nonetheless, the present study demonstrated that the INCOM scale with a two-factor structure and 10 items presents satisfactory psychometric parameters that support and justify its applicability in Brazil, constituting a useful tool to assess social comparison in both research and clinical contexts.
